# Delayed Transverse Colon Stricture Following Blunt Abdominal Trauma: A Case Report With Diagnostic Dilemma

**DOI:** 10.1155/cris/5291507

**Published:** 2026-10-27

**Authors:** Rita Aghjayan, Oluwaseun Adeyemi, Rana Elsayed, Hapreet Sodhi, Qi Wang, Imran Saeed

**Affiliations:** ^1^ Department of Surgery Saint Peter’s University Hospital New Brunswick New Jersey USA saintpetershcs.com; ^2^ Department of Surgery Rutgers Robert Wood Johnson Medical School New Brunswick New Jersey USA rutgers.edu; ^3^ Department of Laboratory Medicine and Pathology Saint Peter’s University Hospital New Brunswick New Jersey USA saintpetershcs.com

## Abstract

This case report describes a 26‐year‐old woman who developed a delayed transverse colon stricture following a motor vehicle crash (MVC). She was a restrained rear‐seat passenger in a rollover collision and sustained blunt abdominal and back injuries, two right rib fractures, superficial lacerations, and T11–L1 vertebral injury requiring surgical management. Contrast‐enhanced computed tomography (CT) performed after the MVC demonstrated no obvious bowel or solid visceral organ injury. Approximately 3 years later, she developed progressive abdominal pain, cramping, fullness, constipation, and a 10‐pound weight loss. Colonoscopy demonstrated a severe, nontraversable stenosis of the transverse colon. Subsequent CT demonstrated marked stool burden and dilatation of the ascending and proximal transverse colon proximal to the stenotic segment. She later presented to the emergency department with worsening abdominal pain, nausea, and vomiting and was diagnosed with large bowel obstruction secondary to the transverse colon stricture. She underwent robotic‐assisted transverse colon resection with colonic lavage and primary colo‐colonic anastomosis. Histopathologic examination demonstrated moderately active chronic colitis with transmural inflammation, glandular architectural distortion, mucosal ulceration, and cryptitis, raising consideration of inflammatory bowel disease. However, no granulomas or dysplasia were identified. The postoperative course was uncomplicated, and at approximately 2 years of follow‐up, she remained asymptomatic without recurrent gastrointestinal symptoms or other clinical features suggestive of inflammatory bowel disease. This case highlights the diagnostic challenge of distinguishing a delayed posttraumatic colonic stricture from inflammatory bowel disease when chronic inflammatory histopathologic changes are present in a patient with remote blunt abdominal trauma.

## 1. Introduction

Transverse colon strictures are uncommon and are typically associated with inflammatory conditions like Crohn’s disease, ischemia, malignancy, or prior surgical interventions. [1, 2] However, in rare instances, strictures may develop as a delayed complication following blunt abdominal trauma, such as a motor vehicle crash (MVC) [3, 4]. Traumatic injuries to the colon often present acutely as perforations or contusions [5, 6], but the subsequent development of fibrosis and stricture formation is a less frequently encountered phenomenon. In such cases, the stricture has been hypothesized to develop following occult bowel wall injury, mesenteric vascular compromise, or localized ischemia sustained during blunt abdominal trauma [7, 8]. These injuries may impair local blood flow, resulting in chronic inflammation and progressive fibrosis of the colonic wall. Over time, these changes may result in gradual luminal narrowing, which may not become clinically apparent until weeks to months or, rarely, years after the initial incident [8]. Diagnosis can be challenging, as the initial trauma may overshadow the later development of gastrointestinal symptoms, and histopathologic findings may overlap with other conditions, particularly inflammatory bowel disease, making definitive attribution of the stricture difficult. This case report presents a rare case of delayed transverse colon stricture following blunt abdominal trauma and discusses the diagnostic considerations associated with establishing its etiology. This case is reported in accordance with the CAse REport (CARE) guidelines for clinical case reports [9].

## 2. Case Presentation

A 26‐year‐old female presented to the emergency department with worsening epigastric pain, abdominal distension, nausea, and vomiting while undergoing bowel preparation for a planned robotic‐assisted transverse colon resection. Her history dates back approximately 3 years, when she was involved in a rollover MVC as a restrained rear‐seat passenger. She sustained blunt abdominal and back injuries, two right rib fractures, superficial lacerations, and T11–L1 vertebral injury requiring surgical management. Contrast‐enhanced computed tomography (CT) of the chest, abdomen, and pelvis obtained following the MVC demonstrated no acute bowel abnormality or solid visceral organ injury. Representative axial and coronal images through the abdomen showed no bowel wall hematoma, focal colonic wall thickening, luminal narrowing, or pericolonic inflammatory change at the approximate level of the subsequently identified transverse‐colon stricture (Figure 1A,B). Over the ensuing years, she developed intermittent abdominal symptoms, with more prominent abdominal cramping, fullness, constipation, and an ~10‐pound weight loss developing in the months preceding her eventual presentation. Approximately 5 weeks before the emergency department presentation, she underwent outpatient surgical evaluation for these symptoms, which included an abdominopelvic CT scan and a colonoscopy.

**Figure 1:**
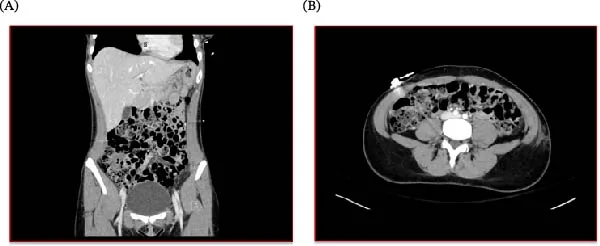
Initial abdominal computed tomography following blunt trauma. (A) Coronal and (B) axial contrast‐enhanced CT images obtained following the motor vehicle crash, at approximately the level of the transverse colon where the stricture was subsequently identified, demonstrating no focal colonic wall thickening, luminal narrowing, or surrounding inflammatory change.

Three weeks before her ED presentation, an abdominopelvic CT and colonoscopy were performed. The contrast‐enhanced CT of the abdomen and pelvis demonstrated a large stool burden within the ascending and proximal transverse colon, with moderate colonic dilatation and mild bowel‐wall thickening (Figure 2A,B). The terminal ileum was also dilated, while the colon distal to the transverse‐colon stenosis was gas‐filled and nondilated. The radiology report noted that the findings could represent regional constipation, although a component of colitis or inflammatory change could not be excluded, and recommended correlation with the colonoscopic findings. Colonoscopy was also performed. The perianal and digital rectal examinations were normal. The colonoscope was advanced to the transverse colon, where a severe stenosis was encountered that could not be traversed, and the examination was therefore incomplete. The stenotic area was biopsied and tattooed. Histopathologic examination of the stricture biopsy demonstrated colonic mucosa with mild architectural distortion, inflamed granulation tissue formation, cryptitis, and reactive glandular changes, interpreted as compatible with an inflammatory colonic polyp. Additional biopsies from the transverse colon, descending colon, sigmoid colon, and rectum demonstrated mucosal edema and intramucosal lymphoid aggregates. In conjunction with the previously demonstrated nontraversable transverse‐colon stenosis, the proximal colonic dilatation and stool burden were clinically concerning for partial large‐bowel obstruction. The patient was subsequently scheduled for robotic‐assisted transverse‐colon resection. While undergoing bowel preparation immediately before the planned operation, she developed worsening abdominal pain, nausea, and vomiting and was unable to tolerate most of the preparation, prompting presentation to the emergency department.

**Figure 2:**
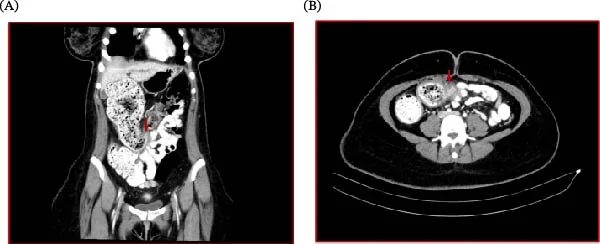
Outpatient contrast‐enhanced computed tomography of the abdomen and pelvis performed approximately 3 weeks prior as part of the abdominal symptoms workup. (A) Coronal image demonstrating moderate dilatation and prominent stool burden within the ascending and proximal transverse colon, with focal bowel‐wall thickening near the distal extent of the dilated segment (red arrow). (B) Axial image demonstrating focal bowel‐wall thickening at the corresponding site (red arrow), with adjacent stool‐filled proximal colon.

At presentation, she described her abdominal pain as acute, sharp, and constant, without identified aggravating or relieving factors. She denied having fever or hematochezia. She reported no tobacco, alcohol, or recreational drug use and had no history of prior abdominal surgery or known inflammatory bowel disease. She also had no history of thromboembolic disease, hypercoagulable disorders, vasculitis, oral contraceptive use, or other recognized risk factors for colonic ischemia. On examination, the patient (height: 154.9 cm, weight: 62 kg, body mass index: 26.1 kg/m^2^) was alert and oriented, with severe abdominal pain rated 10 out of 10. Her blood pressure was 130/89 mmHg, heart rate 93 beats/min, respiratory rate 16 breaths/min, temperature 97.4°F, and oxygen saturation was 100% on room air. On abdominal examination, she was distended and tender, most pronounced in the epigastric region. She was not peritonitic and had no rebound tenderness. Her bowel sounds were hypoactive. Rectal examination revealed normal sphincter tone, with no palpable masses, tenderness, or gross blood. Serum lactate and comprehensive metabolic panel were within normal limits (Table 1). The white blood cell count was elevated at 14.9 × 10^3^/mm^3^ with neutrophil predominance (80.6%). Given her worsening symptoms and inability to tolerate the bowel preparation, repeat contrast‐enhanced CT of the abdomen and pelvis was obtained in the emergency department. The CT demonstrated marked stool‐filled dilatation of the ascending and proximal transverse colon, with focal narrowing and bowel‐wall thickening at the transverse colon corresponding to the previously identified stenotic segment (Figure 3A,B). In the context of her known severe, nontraversable transverse‐colon stenosis and worsening obstructive symptoms, a diagnosis of large‐bowel obstruction secondary to the transverse‐colon stricture was made. She was admitted, maintained nil per oral (nothing by mouth), and a nasogastric tube was inserted. Pain and nausea were managed symptomatically. As she presented to the ED late in the evening and surgical resection had already been planned following her outpatient evaluation, she was scheduled as the first operative case the following morning for urgent transverse colon resection.

**Figure 3:**
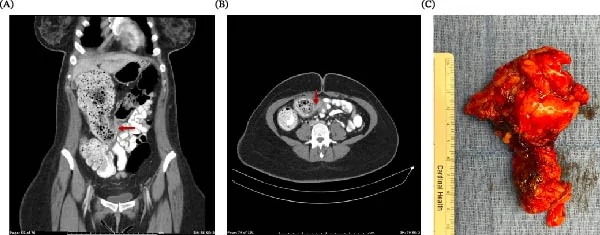
Contrast‐enhanced computed tomography obtained during the acute emergency department presentation and gross operative specimen. (A) Coronal image demonstrating marked stool‐filled dilatation of the proximal colon with abrupt caliber change at the transverse colon (red arrow). (B) Axial image at the corresponding level demonstrating focal narrowing and bowel‐wall thickening of the transverse colon (red arrow). (C) Gross specimen following transverse colon resection demonstrating the stenotic segment.

**Table 1:** Laboratory results during hospital admission.

Test	Day of admission	Postoperative day 1	Reference value
Hematology			
Hemoglobin	15.1	13.1	12.0–16.0 g/dL
Hematocrit	45.7	39.4	42.0%–51.0%
White cell count	14.9	9.9	4.8–10.8 k/uL
Platelet count	258	207	150–400 k/uL
Coagulation			
Prothrombin time	12.1	12.8	10.4–13.7 s
INR	1.09	1.15	0.89–1.11
PTT	30.1	30.8	27.2–35.7 s
General chemistry			
Sodium	136	135	135–145 mEq/L
Potassium	4.6	4.9	3.5–5.0 mEq/L
Chloride	103	102	98–108 mEq/L
Bicarbonate	17 (low value)	22	24–30 mEq/L
Creatinine	0.79	0.64	0.5–1.5 mg/dL
Blood urea nitrogen	10	10	6.0–20.0 mg/dL
Protein	8.8	6.7	6.0–8.0 g/dL
Albumin	5.3	4.2	3.5–5.0 g/dL
Alkaline phosphatase	92	70	56–119 unit/L
ALT	13	9	5–40 unit/L
AST	26	25	9–48 unit/L
Bilirubin (total)	1.0	0.8	0.2–1.2 mg/dL
Lactic acid	2.0	—	0.5–2.0 mmol/L

Abbreviations: ALT, alanine aminotransferase; AST, aspartate aminotransferase; INR, international normalized ratio; PTT, partial thromboplastin time.

The patient underwent robotic‐assisted transverse colon resection with primary anastomosis and lysis of adhesions. On abdominal exploration, moderate adhesions were identified between the transverse colon and the abdominal wall and between small‐bowel loops and the transverse colon. These adhesions were carefully lysed. The transverse colon was mobilized, and a discrete stricture was identified in the mid‐transverse colon at the site of the previously placed endoscopic tattoo. The colon distal to the stricture appeared healthy. Mesenteric windows were created proximal and distal to the stricture, and the involved segment of the transverse colon was divided and resected (Figure 3C). The gastrocolic ligament was opened toward the hepatic flexure to facilitate mobilization of the proximal transverse colon. No gross mesenteric hematoma, ischemic change, or other mesenteric abnormality was observed. The mobilized colon was then exteriorized, and a large amount of solid stool was encountered within the proximal and distal colon. Colonic lavage was performed to clear the bowel. With the proximal and distal bowel ends adequately mobilized and exteriorized, an extracorporeal end‐to‐end colo‐colonic anastomosis was performed. The procedure was completed without any adverse intraoperative event.

The resected specimen was submitted to the hospital pathology department for routine histopathologic examination. Representative sections of the specimen were processed and examined on hematoxylin and eosin (H&E)‐stained slides. Gross pathologic examination of the resected transverse colon demonstrated a 10‐cm segment with luminal diameter ranging from ~2.5 cm at the narrowed segment to 4.5 cm in the wider segment. Irregular hemorrhagic lesions were noted at the area of stenosis, while the remaining mucosa demonstrated unremarkable colonic folds. Histopathologic images demonstrated moderately active chronic colitis with dense inflammatory infiltrate (Figure 4A). The inflammatory infiltrate was described as transmural and composed predominantly of plasma cells with admixed lymphocytes and eosinophils. Mild‐to‐moderate distortion of the glandular architecture was present, consistent with chronicity (Figure 4B), with associated mucosal ulceration (Figure 4C) and focal cryptitis (Figure 4D). No granulomas or dysplasia were identified. The histopathology report noted that the histologic features raised the possibility of inflammatory bowel disease and recommended clinical correlation.

**Figure 4:**
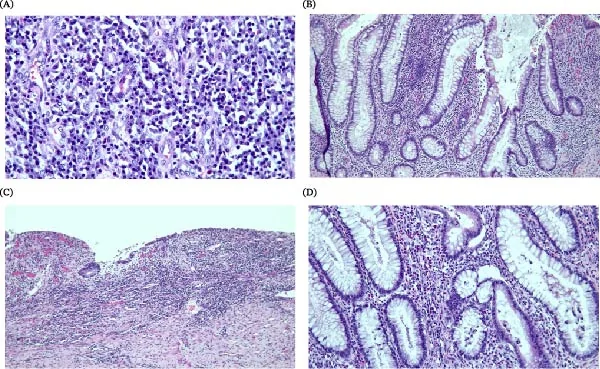
Hematoxylin and eosin (H&E)‐stained sections of the resected transverse colon. (A) High‐power view (×400) demonstrating a dense inflammatory infiltrate composed predominantly of plasma cells with admixed lymphocytes and eosinophils. (B) Low‐power view (×100) showing glandular architectural distortion consistent with chronic inflammation. (C) Low‐power view (×40) demonstrating mucosal ulceration. (D) Intermediate‐power view (×200) demonstrating focal cryptitis.

Postoperative recovery was uneventful, and the patient was discharged home on postoperative day 2 after achieving optimal pain control and a return of bowel function. At approximately 2 years of follow‐up, the patient remained asymptomatic, with no recurrent abdominal pain, altered bowel habits, hematochezia, weight loss, or other gastrointestinal or extraintestinal features suggestive of inflammatory bowel disease. She had not required medical therapy for inflammatory bowel disease or further surgical intervention.

## 3. Discussion

Colonic stricture is an uncommon delayed complication of blunt abdominal trauma, reported in ~3%–5% of cases, with the descending colon most frequently affected [10, 11]. Delayed posttraumatic strictures are not confined to the colon and have also been reported in the small bowel, particularly the ileum, where mesenteric injury, ischemia, and subsequent fibrosis are similarly implicated in their pathogenesis [12–14]. Posttraumatic strictures of the transverse colon are considerably less commonly reported, reflecting differences in colonic mobility and anatomic fixation. The relatively fixed segments of colon—ascending and descending colon—are more susceptible to compression, shearing, and mesenteric vascular injury during rapid deceleration [15], whereas the greater mobility of much of the transverse colon permits displacement and reduces the transmission of traumatic forces [5]. Nevertheless, the transverse colon remains vulnerable to direct compression against the posterior abdominal structures or injury to its mesenteric blood supply. Blunt bowel and mesenteric injuries can result in contusion, mural hematoma, mesenteric injury, ischemia, and, rarely, delayed fibrosis and stricture formation [15–17]. However, trauma represents only one potential cause of a colonic stricture. Other important etiologies include inflammatory bowel disease, ischemic injury, malignancy, and postoperative or anastomotic strictures [18, 19]. For this case, the remote history of blunt abdominal trauma suggested a possible posttraumatic etiology, whereas the chronic inflammatory changes identified on histopathologic examination raised inflammatory bowel disease as an important competing diagnosis. Distinguishing between these etiologies therefore represents the central diagnostic challenge of this case.

Several features of this case support a posttraumatic etiology. The lesion was a discrete, localized stricture of the transverse colon in a young patient with a history of substantial blunt abdominal trauma and no prior abdominal surgery or known gastrointestinal disease. Delayed posttraumatic strictures are thought to result from an initially occult mural or mesenteric injury that produces localized ischemia and inflammation, followed by progressive fibrosis and luminal narrowing [4, 20]. Importantly, the abdominal CT obtained immediately after the MVC showed no bowel abnormality. Although this finding argues against a major acute bowel injury, it does not necessarily exclude a subtle mural or mesenteric injury that was radiographically occult at the time of trauma. Of note, up to 40% of confirmed bowel and mesenteric injuries are missed on a trauma abdominal CT [21]. Additionally, the interval between blunt abdominal trauma and the clinical manifestation of a posttraumatic bowel stricture is variable, with cases presenting weeks, months, or even years after the initial injury [4, 20, 22].

Inflammatory bowel disease, particularly Crohn disease, represents an important competing diagnosis. Histopathologic examination of the resected transverse colon provided the strongest evidence supporting IBD. The histopathologic images demonstrated moderately active chronic colitis with cryptitis, glandular architectural distortion, mucosal ulceration, and reported transmural inflammation—findings that can occur in Crohn disease [23]. In the presence of a colonic stricture, the histopathology findings support Crohn’s disease as a diagnostic consideration [24]. Although no granulomas were identified, their absence does not exclude Crohn disease [23, 24]. However, preoperative biopsies obtained during colonoscopy did not establish IBD. The transverse‐colon biopsy demonstrated edematous colonic mucosa with lymphoid aggregates, while biopsies from the sigmoid colon and rectum similarly demonstrated edema and intramucosal lymphoid aggregates. Of note, chronic ischemic injury can also progress to fibrosis and stricture formation and may produce chronic inflammatory and architectural abnormalities that overlap with IBD [25, 26]. For this index case, a plausible mechanism would be an occult mural injury sustained during the MVC, followed by localized ischemia, chronic inflammation, fibrosis, and progressive stenosis [10, 14]. Thus, although the histopathology of the resected transverse colon raises concern for Crohn disease, the findings are not specific enough to distinguish IBD from a localized chronic ischemic process.

While the absence of a demonstrable bowel or mesenteric injury on the initial posttrauma abdominal CT weakens the evidence of a trauma‐induced stricture, several clinical and operative features similarly weaken an underlying IBD phenotype as the possible etiology. Before development of the stricture‐related symptoms, the patient had no history of chronic or recurrent diarrhea, hematochezia, perianal disease, or previously diagnosed gastrointestinal inflammatory disease, and there was no family history of IBD. She did not smoke and had no documented history of extraintestinal manifestations associated with IBD, including inflammatory arthritis or spondyloarthropathy, ocular inflammation, hepatobiliary disease, erythema nodosum, pyoderma gangrenosum, or recurrent oral lesions [27, 28]. Although she subsequently developed abdominal pain, constipation, fullness, and weight loss, these symptoms might have occurred in the setting of a progressively obstructing focal colonic stricture rather than a preceding relapsing inflammatory gastrointestinal syndrome. Crohn disease characteristically may demonstrate discontinuous involved segments, aphthous ulcers progressing to deep linear or serpiginous ulceration, cobblestoning, fistulae, and, in established disease, creeping mesenteric fat [29]. At surgery, the abnormality was localized to the tattooed mid‐transverse‐colon segment, the distal colon appeared grossly healthy, and no fistulae, gross mesenteric inflammatory changes, or additional areas of gross bowel involvement were documented. Following resection, the patient remained clinically well for approximately 2 years without IBD‐directed therapy, recurrent abdominal pain, altered bowel habits, hematochezia, weight loss, or newly recognized extraintestinal manifestations. These findings reduce the clinical support for an established Crohn phenotype but do not exclude isolated Crohn’s disease or the subsequent emergence of a more definitive IBD phenotype.

Determining the etiology of the stricture also has implications for long‐term management. Segmental resection with primary anastomosis was appropriate in this patient because she presented with a localized, severe, nontraversable stricture complicated by large bowel obstruction. If the lesion represents an isolated posttraumatic stricture, complete resection of the affected segment may be definitive, with no disease‐specific medical therapy required [10]. In contrast, if the stricture represents an initial manifestation of Crohn disease, disease recurrence or progression may occur despite successful resection of the index lesion [30]. In patients with Crohn‐related strictures, future management depends on the location, length, and inflammatory or fibrotic characteristics of the lesion and may include medical therapy, endoscopic balloon dilation, stricturoplasty, or resection [24, 31]. Bowel‐preserving approaches are particularly important in patients requiring repeated interventions because cumulative intestinal resection increases the risk of short‐bowel syndrome [32]. In the present patient, the absence of recurrent gastrointestinal symptoms or additional strictures approximately 2 years after resection is reassuring and provides some clinical support for an isolated process. However, this duration of follow‐up does not definitively exclude a future diagnosis of Crohn disease. Continued clinical follow‐up is therefore warranted.

This case report has its limitations. First, we could not definitively establish the etiology of the colonic stricture. No bowel or mesenteric injury was identified on the initial posttrauma CT, although subtle injuries may have been radiographically occult, and the histopathologic findings could not reliably distinguish a chronic posttraumatic or ischemic process from Crohn disease. Second, additional IBD‐directed evaluation, including magnetic resonance enterography and serologic or stool inflammatory markers, was not performed, limiting further assessment for inflammatory disease outside the resected segment. Third, although the patient remained clinically well for approximately 2 years without IBD‐directed therapy, this follow‐up period cannot definitively exclude the subsequent emergence of Crohn disease. Despite these limitations, this case report is strengthened by the availability of the original trauma imaging, interval cross‐sectional imaging demonstrating the evolution of the colonic abnormality, endoscopic and biopsy findings, operative findings, resection histopathology, assessment of alternative ischemic risk factors, and approximately 2 years of postoperative clinical follow‐up.

This case illustrates the diagnostic challenge presented by a delayed focal colonic stricture in a patient with remote blunt abdominal trauma when the histopathologic findings also raise concern for inflammatory bowel disease. Although the clinical history, focal nature of the lesion, absence of a preceding IBD phenotype, and symptom‐free follow‐up after segmental resection support a possible posttraumatic etiology, the chronic active and transmural inflammatory changes preclude definitive exclusion of IBD. Clinicians evaluating otherwise unexplained colonic stenosis should therefore consider remote abdominal trauma in the differential diagnosis while simultaneously excluding more common inflammatory, ischemic, neoplastic, and postoperative causes.

## Author Contributions

Conceptualization: Rita Aghjayan, Oluwaseun Adeyemi, and Imran Saeed. Data curation: Rita Aghjayan, Oluwaseun Adeyemi, Qi Wang, and Imran Saeed. Writing – original draft: Rita Aghjayan, Oluwaseun Adeyemi, Rana Elsayed, and Hapreet Sodhi. Project administration, supervision: Oluwaseun Adeyemi and Imran Saeed. Writing – review and editing: Rita Aghjayan, Oluwaseun Adeyemi, Rana Elsayed, Hapreet Sodhi, Qi Wang, and Imran Saeed.

## Funding

The authors received no financial support for the research, authorship, and/or publication of this article.

## Ethics Statement

Our institution does not require ethical approval for reporting individual cases or case series.

## Consent

Written informed consent was obtained for the publication of the information included in this article. Written informed consent for publication was provided by the participant.

## Conflicts of Interest

The authors declare no conflicts of interest.

## Data Availability

The data underlying this case report are available from the corresponding author upon request. The journal may review the data to verify the findings as needed.
